# Protective effects of intercalated disk protein afadin on chronic pressure overload-induced myocardial damage

**DOI:** 10.1038/srep39335

**Published:** 2017-01-03

**Authors:** Dimitar P. Zankov, Akio Shimizu, Miki Tanaka-Okamoto, Jun Miyoshi, Hisakazu Ogita

**Affiliations:** 1 Division of Molecular Medical Biochemistry, Department of Biochemistry and Molecular Biology, Shiga University of Medical Science, Seta Tsukinowa-cho, Otsu, Shiga 520-2192, Japan; 2 Department of Molecular Biology, Osaka Medical Center for Cancer and Cardiovascular Disease, 1-3-3 Nakamichi, Higashinari-ku, Osaka 537-8511, Japan

## Abstract

Adhesive intercellular connections at cardiomyocyte intercalated disks (IDs) support contractile force and maintain structural integrity of the heart muscle. Disturbances of the proteins at IDs deteriorate cardiac function and morphology. An adaptor protein afadin, one of the components of adherens junctions, is expressed ubiquitously including IDs. At present, the precise role of afadin in cardiac physiology or disease is unknown. To explore this, we generated conditional knockout (cKO) mice with cardiomyocyte-targeted deletion of afadin. Afadin cKO mice were born according to the expected Mendelian ratio and have no detectable changes in cardiac phenotype. On the other hand, chronic pressure overload induced by transverse aortic constriction (TAC) caused systolic dysfunction, enhanced fibrogenesis and apoptosis in afadin cKO mice. Afadin deletion increased macrophage infiltration and monocyte chemoattractant protein-1 expression, and suppressed transforming growth factor (TGF) β receptor signaling early after TAC procedure. Afadin also associated with TGFβ receptor I at IDs. Pharmacological antagonist of TGFβ receptor I (SB431542) augmented mononuclear infiltration and fibrosis in the hearts of TAC-operated control mice. In conclusion, afadin is a critical molecule for cardiac protection against chronic pressure overload. The beneficial effects are likely to be a result from modulation of TGFβ receptor signaling pathways by afadin.

Afadin is initially found to be essentially involved in the structure, function, and organization of adherens junctions (AJs). Afadin links intracellular actin filaments and nectins, transmembrane proteins that form homo- or hetero-dimers in *trans*, to establish cell-cell adhesive complex^1^,^2^. Given the fact that cell-cell contact is of fundamental importance in multicellular organisms and the multitude of interacting domains in the native protein structure^3^, it has been found that afadin possesses broad spectrum of functionality ranging from embryonic development to progression of some neoplasms^4^,^5^,^6^. In addition, afadin also acts as an independent signaling molecule to mediate several cellular processes like cell motility or synaptic plasticity in neurons^7^,^8^.

Since its discovery, afadin has been detected in the cardiac vasculature (endothelium) and myocytes^2^. In cardiomyocytes, it is located at the subcellular structures with central importance for the electrical and mechanical activity of the myocardium: the intercalated disks (IDs). Traditionally, IDs are described as assembly of AJs (the afadin locus), desmosomes, and gap junctions that assure contact with actin filaments, intermediate filaments and cytosolic communication, respectively^9^. Recently, complex interactions and hybrid molecular complexes are depicted at IDs, creating the term “area composita” instead of separating AJs and desmosomes. Those mixed junctional aggregates show that entire IDs might be considered one functional unit^10^. Animal models and investigations of human diseases have demonstrated that aberrant function of ID proteins (e.g., N-cadherin, desmosomal components, connexins) are related to potentially lethal diseases like dilated cardiomyopathy and arrhythmogenic right ventricular cardiomyopathy^11^. Sophisticated ID structure also defends the heart during chronic stress^12^. At present, the precise significance of afadin for the cardiomyocyte function is unknown. Given its critical localization, the understanding of the protein role may add to the knowledge of ID machinery in physiology or disease.

Chronic pressure overload is a frequent risk factor for cardiac morbidity and mortality. The overload is most often a direct result of increased systemic arterial resistance as in the various forms of hypertension or mechanical obstacle to the blood flow as in aortic stenosis or coarctation. This causes left ventricular (LV) hypertrophy, global cardiac remodeling and cardiac fibrosis, and if untreated, tends to progress to end-stage heart failure. Chronic pressure overload-related remodeling is a result of multiple pathophysiological mechanisms that occur in parallel, and affects profoundly all cellular constituents and intercellular matrix as well as electrical and pump function of the heart^13^,^14^. Myriad of signaling mechanisms and cellular events are described in chronic pressure overload-associated cardiac remodeling with the hope that suitable targets that may improve clinical output could be found^15^.

Because of severity and prevalence, LV hypertrophy (independent cardiac risk factor), fibrosis and failure are main topics in clinical and basic research^16^. Therapeutically, improvement/reverse of those pathophysiological conditions is often problematic due to the complexity and still incomplete understanding of underlying mechanisms^17^,^18^. In this study, we investigate the function of afadin in the myocardium using a mouse model. Selective deletion of the protein in cardiomyocytes causes progressive cardiac dysfunction, enhanced fibrosis and apoptosis under the condition of chronic stress produced by chronic pressure overload. Our findings suggest that afadin might be considered as a new target in the management of chronic pressure overload-associated cardiac fibrosis, apoptosis and dysfunction.

## Results

### Characteristics of afadin cKO mice

The proportion of born afadin cKO mice complied with the Mendel’s laws and their appearance was undistinguishable from the littermates. For the period of experimental work, we have been breeding homozygous afadin-floxed with afadin-floxed/Myh6-Cre mice to produce half of the offspring as afadin cKO. From total of 263 mice, afadin cKO genotype has been found in 126 (47.9%); 131 of the mice were female. To confirm actual deletion of afadin in afadin cKO mice, we obtained protein samples from hearts, lungs, livers, spleens and kidneys, and tested the expression levels of afadin. In afadin cKO hearts, the band density in Western blots has been reduced markedly, but did not fade completely, because of the protein from non-cardiomyocytes (Supplementary Fig. S1a). In all other protein samples, afadin retained similar expression. Immunohistochemical staining of heart cryosections demonstrated typical localization of afadin in control (Supplementary Fig. S1b, white arrows) and lack of fluorescence at IDs in afadin cKO mice. Afadin in non-cardiomyocytes was still visualized as lines parallel with the long axis of the cardiomyocytes in afadin cKO mice (Fig. S1b, yellow arrowheads). We also compared gross cardiac morphology in hematoxylin and eosin (HE) stained sections (Supplementary Fig. S1c). Control and afadin cKO mice displayed similar pattern: there was no distinction in the histological characteristics of cardiomyocytes, cellular content of interstitium, or vascular properties. Further, there was no significant difference between the *in vivo* parameters of blood pressure, and cardiac function, chambers and wall dimensions in the control and afadin cKO mice at 8 weeks of age (Supplementary Table S1). Thus, myocardial afadin appears to be not obligatory for cardiac embryonic development and of imperceptible importance for cardiac structures and function at physiological conditions.

### Severe cardiac dysfunction in afadin cKO hearts by transverse aortic constriction

**Figure 1:**
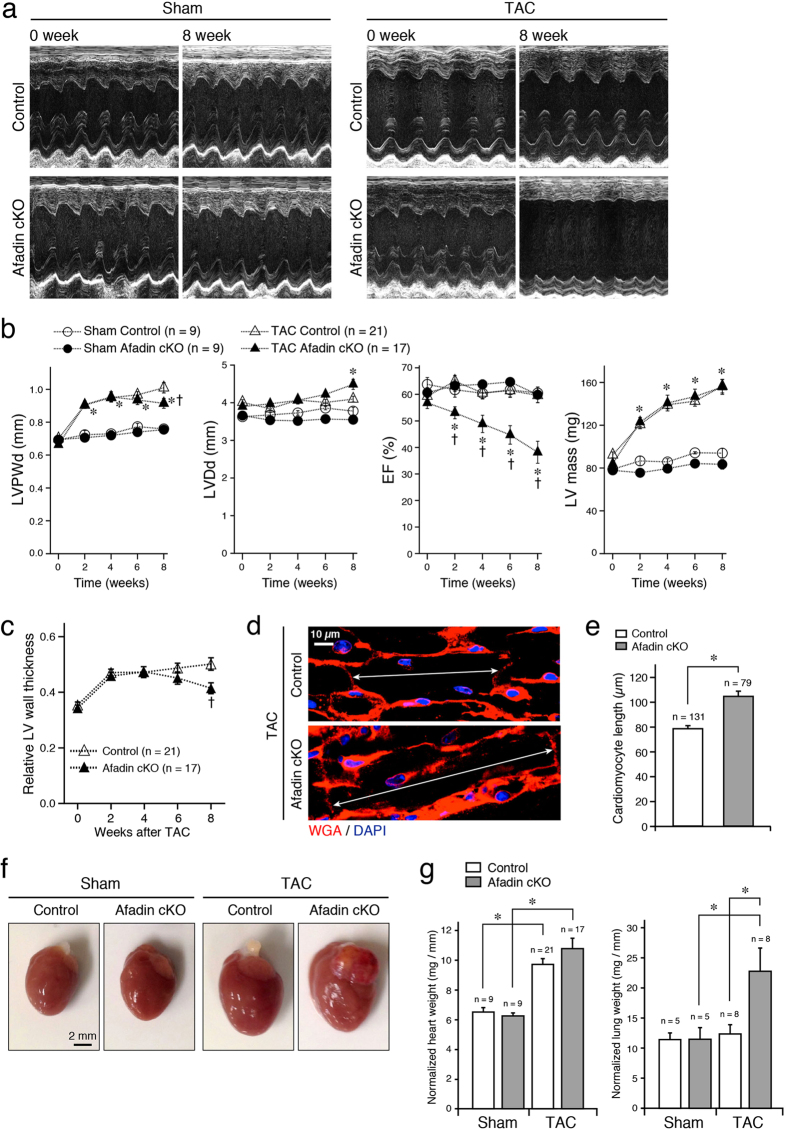
*In vivo* monitoring and morphometric evaluation of hearts and lungs in TAC-challenged mice. (**a**) Images of M-mode echocardiography in sham- or TAC-operated mice before (0 week) and after 8 weeks of observation. (**b**) Summary graphs of LV echocardiographic parameters before, 2, 4, 6, and 8 weeks after the sham or TAC procedure. LVPWd: LV posterior wall diastolic thickness, LVDd: LV diastolic diameter, EF: ejection fraction. *p < 0.05 vs sham-operated mice, ^†^p < 0.05 vs TAC-operated control mice. (**c**) Summary graph of relative LV wall thickness in TAC-operated mice calculated as: 2 × LVPWd (mm)/LVDd (mm). *p < 0.05 vs sham-operated mice, ^†^p < 0.05 vs TAC-operated control mice. (**d**) Staining of cell membranes with wheat germ agglutinin (WGA: red) to measure the cardiomyocyte length (double arrows). DAPI (blue) staining for the nuclei. (**e**) Summary graph of cardiomyocyte lengths. *p < 0.05. (**f**) Photographs demonstrating enlargement of control and afadin cKO hearts after establishing TAC for 8 weeks in comparison to the sham treatment. (**g**) Summary graphs of heart and lung weights (mg) normalized to the tibia length (mm). *p < 0.05.

To explore the significance of cardiac afadin in pathological conditions, we generated chronic pressure overload stress model by transverse aortic constriction (TAC) procedure, and followed the mice for 8 weeks. Figure 1a presents characteristic M-mode echocardiographic images of sham- and TAC-operated mice, respectively, comparing initial (no treatment, 0 week) and concluding (8 week after the intervention) LV status. There was no significant change of LV parameters between control and afadin cKO mice in the sham-operated group (Fig. 1b). In the first 4 weeks after TAC procedure, both control and afadin cKO mice progressively developed LV hypertrophy marked by wall thickening, increase in LV mass and unchanged cavity diameter (concentric hypertrophy), although LV pump function declined in afadin cKO mice (Fig. 1b). In the second 4 weeks after TAC, control mice maintained normal LV systolic function in sharp contrast to afadin cKO mice that evolved to severe LV systolic dysfunction, chamber dilation and reduction of LV wall thickness (eccentric hypertrophy) (Fig. 1b). This time course of LV wall and cavity dynamics is presented clearly by the relative wall thickness calculation and increased length of cardiomyocytes in afadin cKO hearts compared to control at the end of observation period (Fig. 1c–e). Morphometric quantifications of all studied hearts demonstrated that banding of the aorta for 8 weeks resulted in obvious cardiac hypertrophy (Fig. 1f). Normalized heart weight in the sham-operated group was similar between control and afadin cKO mice (Fig. 1g). In the TAC-operated group, normalized heart weight significantly increased to a similar extent in both control and afadin cKO mice. On the other hand, considerable growth of normalized lung weight, an indicator of pulmonary congestion as a result of LV pump failure, was observed only in TAC-operated afadin cKO mice (Fig. 1g).

**Figure 2:**
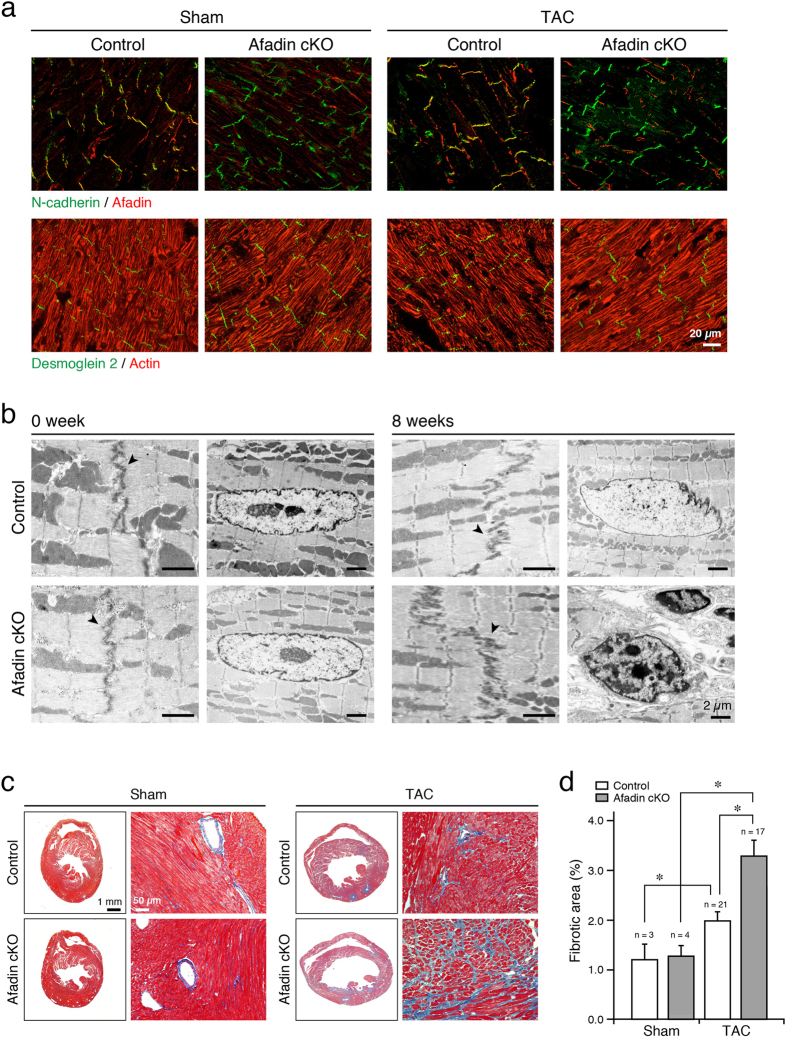
Morphology of IDs and quantification of fibrotic accumulation in TAC-operated mice. (**a**) Co-immunostaining of AJ components N-cadherin (green) and afadin (red), or desmosomal cadherin, desmoglein 2 (green), and actin filaments (red) in cardiac sections following 8-week sham or TAC operation. (**b**) Electron microscopic images demonstrating the structure of IDs (arrowheads) and subcellular components of cardiomyocytes. (**c**) Images of cardiac sections stained with Masson’s trichrome 8 weeks after operation. Left: lower magnification (2x) of the whole heart sections, Right: higher magnification (20x). Blue staining: interstitial fibrosis. (**d**) Summary graph of fibrosis abundance in histological preparations. *p < 0.05.

Genetic modifications aiming at selective loss of adhesive proteins in cardiac AJs or desmosomes disrupt the structure of IDs either in physiological state or during stress^19^,^20^. In addition, some components of junctional complexes are also dislocated or missing. Afadin supports AJs through its association with actin filaments. Although the chronic pressure overload by TAC remodeled afadin-deleted hearts, confocal images of IDs did not show structural alterations, and AJ or desmosomal constituents (N-cadherin and desmoglein 2, respectively) appeared to be not displaced (Fig. 2a). Overall structure of actin filaments was also unaffected. Electron microscopy at basic conditions (0 week, before TAC procedure) showed identical IDs, and sarcomeric and nuclear morphology in control and afadin cKO hearts (Fig. 2b). In both types of mice, myocardial remodeling induced by 8 weeks TAC widened ID structures to a similar extent, which is consistent with the previous report^21^. In afadin cKO hearts, however, nuclei with condensed chromatin and altered shape (characteristics of apoptosis) were frequently observed. Prohypertrophic and fibrogenic signaling is interwoven in the course of cardiac remodeling induced by pressure overload, making fibrosis and hypertrophy coincide frequently^13^. We evaluated fibrotic accumulation in sham and TAC hearts by analyzing cardiac sections stained with Masson’s trichrome. Contrary to sham hearts, we found increased interstitial and perivascular fibrosis in the sections of TAC-operated mice, especially in afadin cKO hearts (Fig. 2c,d). Thus, along with the reduction of LV pump function, afadin cKO mice tended to enhance myocardial fibrogenesis when exposed to chronic pressure overload.

### Cellular responses in the course of TAC-induced chronic pressure overload

**Figure 3:**
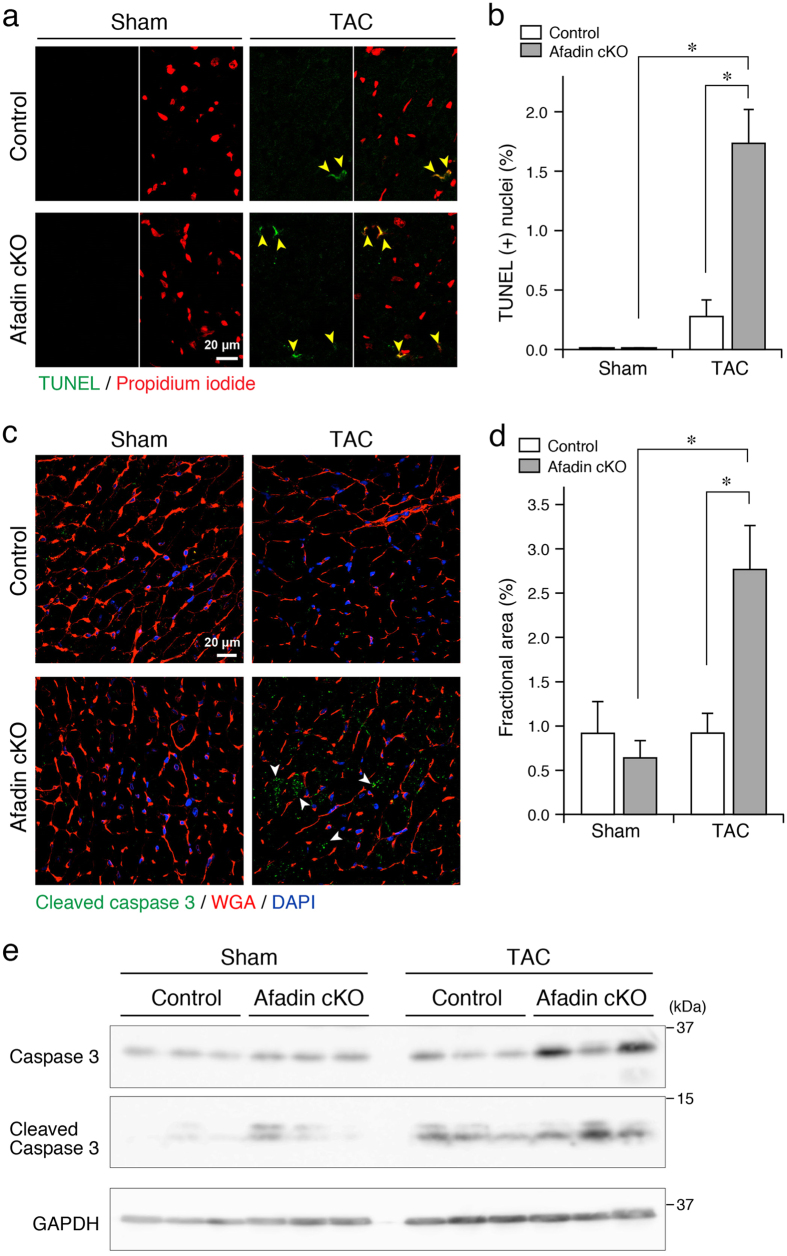
Cellular markers for apoptosis in TAC-exposed mouse hearts. (**a**) Confocal images showing TUNEL-positive apoptotic nuclei (green) among the scanned nuclei stained with propidium iodide (red) in all groups. Yellow arrowheads: TUNEL-positive cells. (**b**) Summary graph presenting percentage of TUNEL-positive nuclei, which was estimated per 1000 nuclei in each group. *p < 0.05. (**c**) Immunostaining of cleaved caspase 3 (green). Wheat germ agglutinin (WGA: red) staining for the surface membrane of cardiomyocytes, and DAPI (blue) staining for the nuclei. White arrowheads: cleaved caspase 3-positive areas. (**d**) Summary graph presenting percentage of cleaved caspase 3-positive area. *p < 0.05. (**e**) Western blots for detection of cleaved caspase 3 in the protein samples from sham- and TAC-operated hearts for 8 weeks (n = 3 in each group). GAPDH: loading control.

Enhanced apoptosis of cardiomyocytes is one of the mechanisms worsening cardiac function during pressure overload^22^. We analyzed the level of apoptosis by the means of terminal deoxynucleotidyl transferase-mediated dUTP nick-end-labeling (TUNEL) reaction and immunostaining for cleaved caspase 3 to detect DNA fragmentation in apoptotic nuclei and activated apoptotic signaling, respectively. DNA segmentation, a hallmark of apoptotic cell death, was hardly found in the sham-operated group (Fig. 3a,b). After the TAC procedure, TUNEL-positive nuclei were remarkably increased in afadin cKO mice comparing with control mice. In agreement with these results, TAC significantly enhanced immunostaining intensity of cleaved caspase 3 in afadin cKO mice (Fig. 3c,d). In Western blots using cardiac samples, band densities of cleaved caspase 3 were low after sham operation, while TAC procedure increased the band density, especially in afadin cKO mice (Fig. 3e). These data suggest that afadin may suppress augmented apoptotic response by TAC-induced pressure overload in the hearts.

**Figure 4:**
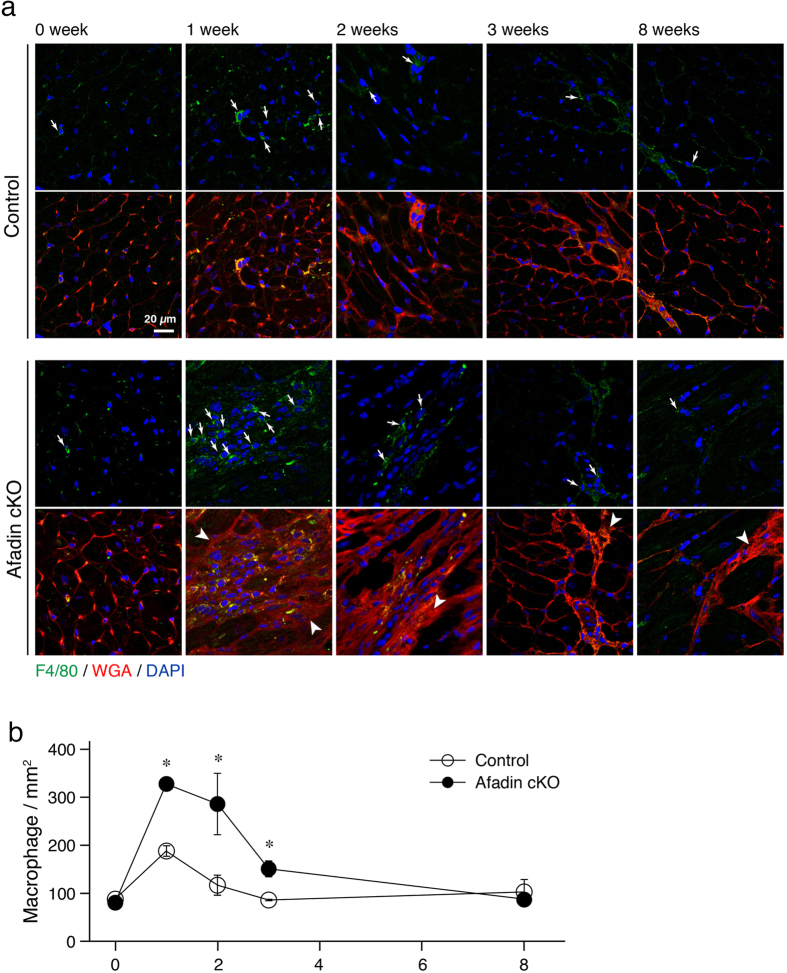
Inhibition of early inflammatory reaction by afadin after TAC operation. (**a**) Confocal images demonstrating infiltration of myocardium with F4/80-positive cells (green) in the time series of untreated (0 week), 1, 2, 3, and 8 weeks exposure to chronic pressure overload induced by TAC procedure. Wheat germ agglutinin (WGA: red) staining for the cell membranes, and DAPI (blue) staining for the nuclei. Arrows: F4/80-positive infiltrating cells, Arrowheads: disturbed myocardial structure. (**b**) Summary graph for quantification of F4/80-positive cells. *p < 0.05 vs control.

It is acknowledged that immune cell proliferation is one of the responses in chronic stress-challenged myocardium that promotes collagen synthesis^23^. In TAC mice, the inflammatory reaction develops early after the procedure, consists mainly of monocytes/macrophage, and is gradually waning^24^. We analyzed the extent of inflammation in mouse hearts exposed to TAC for 1, 2, 3 and 8 weeks by detecting the cells positive for F4/80, an adhesive receptor specifically expressed in mature macrophage^25^. Consecutive confocal images demonstrated that the number of resident macrophages in control and afadin cKO mice was low and identical before TAC (Fig. 4). We observed higher increase of macrophages in afadin cKO mice after 1, 2 and 3 weeks after TAC, compared with control TAC-operated mice. It was also noticeable that in the areas of macrophage clustering, myocardial structure in afadin cKO mice was disturbed, reflecting the progress of cardiac remodeling.

### Transforming growth factor β receptor-mediated signaling mechanisms during TAC-induced chronic pressure overload

**Figure 5:**
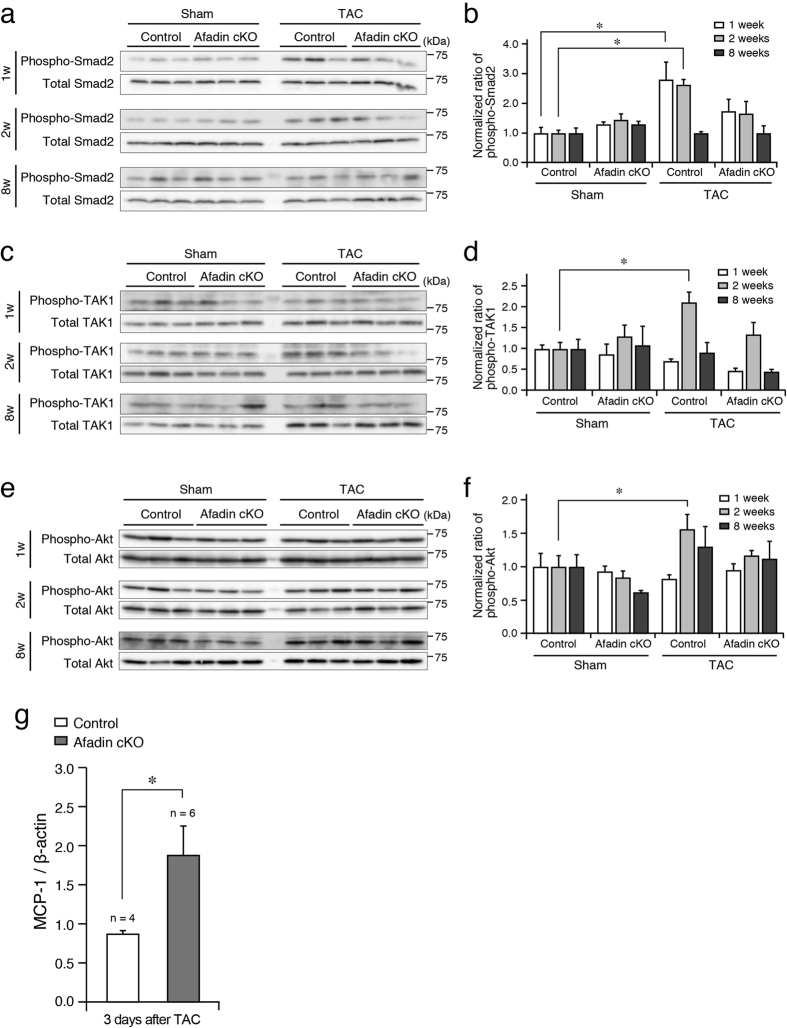
Relationship between afadin and TGFβ receptor-related signaling pathways during chronic pressure overload. (**a**, **c** and **e**) Western blots showing the degree of phosphorylated and total proteins of Smad2 (**a**), TAK1 (**c**) and Akt (**e**) from mouse hearts after 1, 2, and 8 weeks of sham and TAC operation (n = 3 in each group). (**b**,**d** and **f**) Summary graphs for quantification of phosphorylated/total protein band density ratios of of Smad2 (**b**), TAK1 (**d**) and Akt (**f**), which were normalized to the samples from control sham-operated mice. (**g**) Comparison of MCP-1 mRNA levels in the control and afadin cKO cardiomyocytes 3 days after TAC procedure. *p < 0.05.

**Figure 6:**
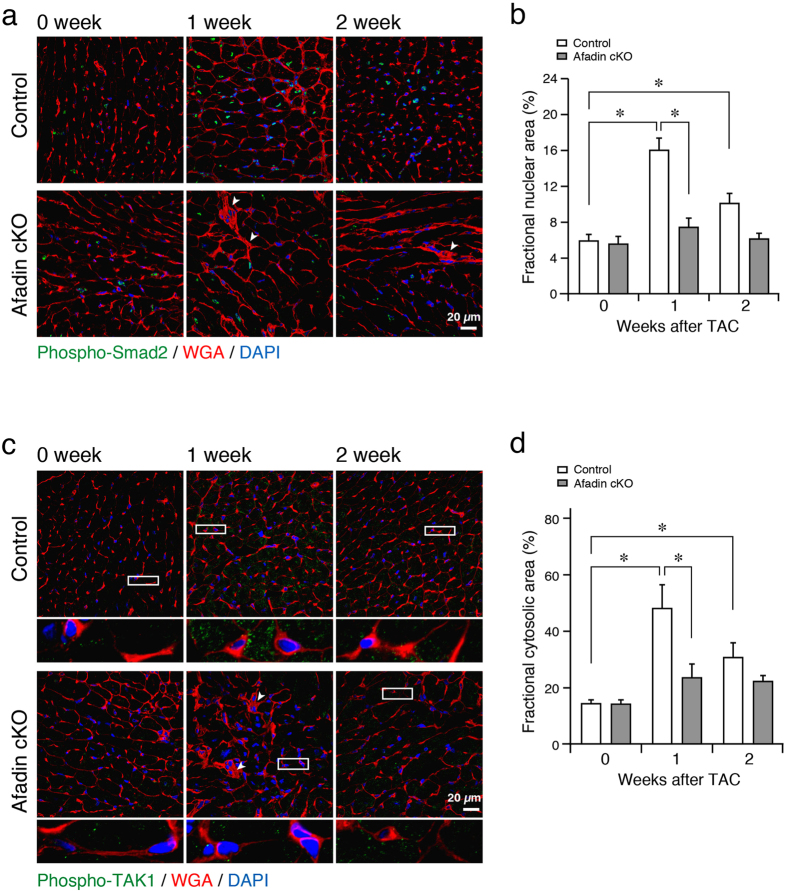
Phosphorylated Smad2 and TAK1 after TAC procedure in control and afadin cKO mice. (**a** and **c**) Immunostaining of activated (phosphorylated) Smad2 (**a**) and TAK1 (**c**) in the control and afadin cKO hearts during pressure overload challenge. Images below the respective phospho-TAK1 photos showing magnification of depicted rectangular areas. Wheat germ agglutinin (WGA: red) staining for the cell membranes, and DAPI (blue) staining for the nuclei. Arrowheads: disturbed myocardial structure. (**b** and **d**) Summary graphs for percentage of phospho-Smad2-positive nuclear area (**b**) and phopsho-TAK1-positive cytosolic area (**d**), which were estimated per 1000 nuclei and cells in each group. *p < 0.05.

Transforming growth factor (TGF) β signaling is one of the key factors in stress-related cardiac remodeling. TGFβ pathways are obligatory for pressure overload-produced cardiac hypertrophy, fibrosis and dysfunction^26^,^27^. On the other hand, TGFβ signaling is versatile and context-dependent, resulting in opposing effects^28^,^29^. We evaluated the activation (phosphorylation) of Smad2 and TGFβ activated kinase (TAK) 1, signaling molecules downstream of TGFβ receptor, by Western blot using protein samples from the mouse hearts. In control hearts, phosphorylation of Smad2 was elevated at 1 and 2 weeks after banding of the aorta (Fig. 5a,b). Contrary, in afadin cKO hearts, chronic pressure overload did not significantly enhance Smad2 phosphorylation. Phosho-TAK1 followed similar to Smad2 pattern: increase in control, but not in afadin cKO TAC hearts (Fig. 5c,d). We also performed immunohistochemistry to observe the above Smad2 and TAK1 phosphorylation pattern specifically in cardiomyocytes. Similar to the results from Western blots, significant increases in Smad2 phosphorylation, which was visible in the nuclei of cardiomyocytes, and TAK1 phosphorylation, which was located in the cytosolic area with dot-like appearance, were detected only in the control hearts 1 and 2 weeks after TAC procedure (Fig. 6). Akt is also known as a modulator in the development of cardiac remodeling, and acts downstream of TGFβ receptor^30^,^31^. We have previously reported that afadin activates Akt, leading to protection against apoptosis-inducing stimulations^32^. Western blots in control mice demonstrated that phosphorylated Akt was increased 2 weeks after TAC procedure, but afadin cKO mice did not alter the Akt level (Fig. 5e,f). Collectively, these findings suggest that myocardial afadin is an essential step for transduction of TGFβ receptor-related signaling early after establishing pressure overload by TAC procedure.

We further examined how macrophage accumulation was enhanced in afadin cKO hearts at the early phase after TAC. Although it may be possible that cardiac injury such as increased apoptosis causes the promotion of macrophage infiltration in the heart, the levels of apoptosis observed by TUNEL and cleaved caspase 3 immunostaining assays were low in both control and afadin cKO hearts during 3 weeks after TAC, compared with the level in afadin cKO hearts (Supplementary Fig. S2), suggesting that macrophage accumulation in the heart may be independent of enhanced apoptosis in this study. Next, because one of the critical factors recruiting macrophages in the course of inflammatory responses is monocyte chemoattractant protein 1 (MCP-1), its expression immediately (3 days) after TAC was analyzed by quantitative PCR using isolated cardiomyocytes to specifically certify the importance of myocardial afadin deletion for the recruitment of macrophages in the heart. MCP-1 is synthesized in cardiomyocytes and its expression is reported to be negatively regulated by TGFβ^33^,^34^. Compared with control cardiomyocytes, MCP-1 mRNA in afadin cKO cardiomyocytes is significantly upregulated (Fig. 5g). These results may explain (at least partially) that macrophage accumulation early after TAC is not due to the secondary effects induced by cardiac injury, but that the increase in MCP-1 immediately after TAC as a result of afadin-modulated regulation of TGFβ signaling in cardiomyocytes is involved in the promotion of macrophage infiltration in the heart.

**Figure 7:**
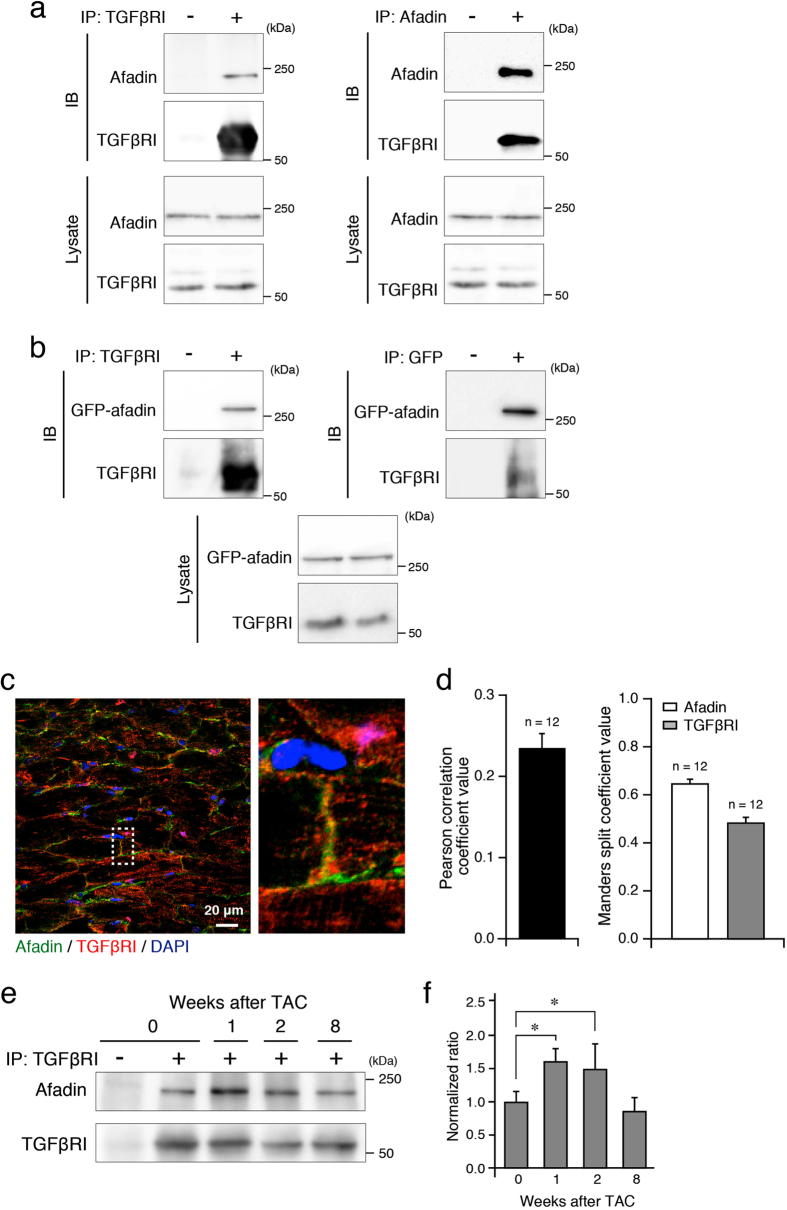
Association of afadin and TGFβ receptor I. (**a** and **b**) Immunoprecipitation using indicated antibodies to examine the association of afadin and TGFβ receptor I (TGFβRI) in the samples from the control mouse hearts (**a**) and from COS7 cells transfected with GFP-afadin (**b**). IP: immunoprecipitation, IB: immunoblot for immunoprecipitated samples. Asterisks: IgG heavy chain. The images are representative from at least 3 separate experiments. (**c**) Co-immunostaining of afadin (green) and TGFβRI (red). Image on the right side: the enlargement of area surrounded by dotted rectangle. DAPI (blue) staining for the nuclei. (**d**) Mean values of Pearson correlation coefficient and Manders split coefficients obtained from confocal images (**c**) in order to demonstrate positive co-localization between afadin and TGFβ receptor I in control hearts. (**e**) Immunoprecipitation experiments to evaluate the time course of afadin-TGFβRI association during chronic pressure overload. (**f**) Summary graph of the afadin-TGFβRI association ratio, which were normalized to the sample before (0 week) TAC (n = 5 in each group). *p < 0.05.

Pressure overload in afadin cKO mice subjected to banding of the aorta induced enhanced fibrosis, apoptosis, macrophage infiltration and heart failure that coincided with depressed TGFβ signaling (Figs 1, 2, 3, 4, 5 and 6). In order to verify the association between cardiac afadin and TGFβ receptor, we performed immunoprecipitation and co-immunostaining experiments. The immunoprecipitation experiments detected the association of afadin and TGFβ receptor I in both mouse hearts and ectopically expressed cell systems (Fig. 7a,b). We also found the co-localization of afadin and TGFβ receptor I at IDs in co-immunostaining experiments (Fig. 7c,d). Moreover, association of TGFβ receptor I with afadin in the heart of control mice following TAC operation showed a time-dependent increase, resembling dynamics of mononuclear infiltration and TGFβ receptor-related signaling molecules (Fig. 7e,f).

**Figure 8:**
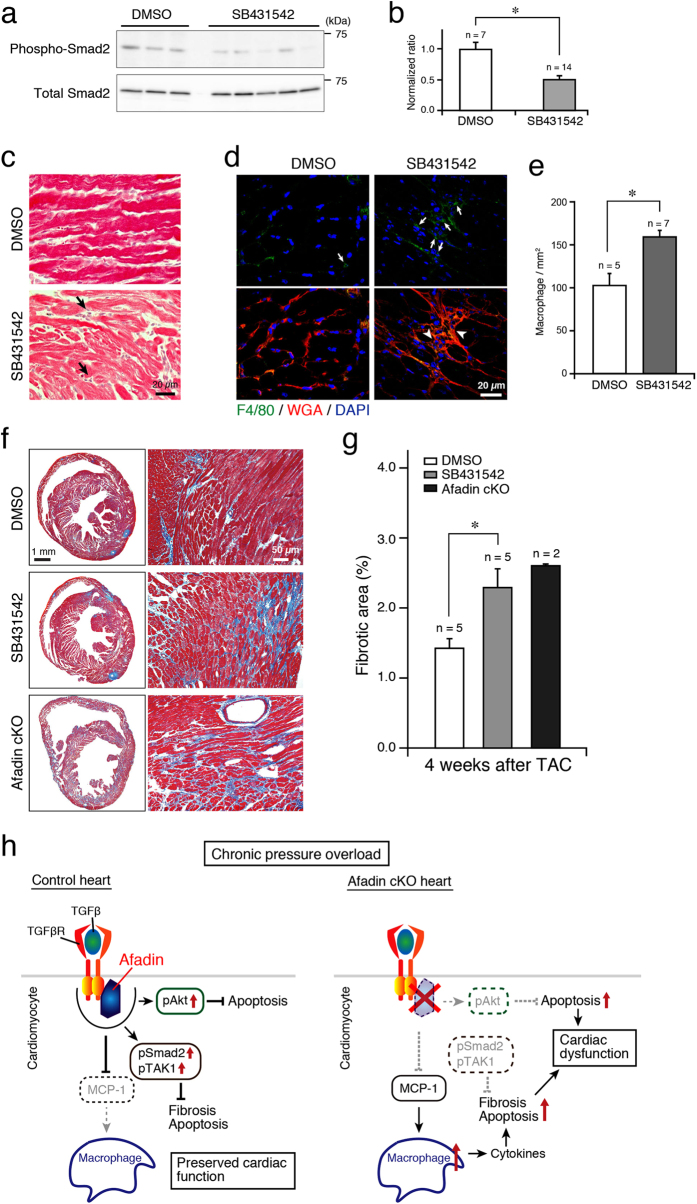
Effect of pharmacological inhibition of TGFβ receptor I by SB431542. (**a**) Western blots of phosphorylated and total Smad2 in the protein samples from control TAC-operated hearts treated with DMSO (negative control) or selective TGFβ receptor I blocker SB431542 for 2 weeks. (**b**) Summary graph of phosphorylated/total Smad2 band density ratios, which were normalized to DMSO-treated mice. *p < 0.05. (**c**) HE-stained cardiac sections of DMSO- and SB431542-treated mice. Arrows: mononuclear infiltration in the interstitium. (**d**) Confocal images demonstrating infiltration of myocardium with F4/80-positive cells (green) in the DMSO- and SB431542-treated hearts. Wheat germ agglutinin (WGA: red) staining for the cell membranes, and DAPI staining for the nuclei. Arrows: F4/80-positive infiltrating cells, Arrowheads: disturbed myocardial structure. (**e**) Summary graph for quantification of F4/80-positive cells. *p < 0.05. (**f**) Images of cardiac sections stained with Masson’s trichrome after 4-week TAC-induced pressure overload. Left: lower magnification (2x) of the whole heart sections, Right: higher magnification (20x). (**g**) Summary graph of stained fibrotic areas in the cardiac sections. (**h**) Cartoon of proposed mechanism of afadin action during pressure overload challenge. Deletion of afadin suppresses TGFβ receptor downstream signaling and affects macrophage recruitment, enhancement of apoptosis, fibrosis and cardiac dysfunction. MCP-1: monocyte chemoattractant protein 1, pAkt: phosphorylated Akt, pSmad2: phosphorylated Smad2, pTAK1: phosphorylated TAK1.

Finally, pharmacological inhibition of TGFβ receptor I was conducted using a selective antagonist SB431542^35^. Administration of SB431542 for 2 weeks in control mice that underwent TAC procedure actually resulted in suppression of Smad2 phosphorylation, compared with that of DMSO (Fig. 8a,b). In SB431542-treated but not DMSO-treated hearts, HE staining showed mononuclear infiltration in the interstitium (Fig. 8c). More specifically, F4/80-positive cells were abundant and disturbed myocardial structure was observed in SB431542-treated mice (Fig. 8d,e). When TAC-operated control mice were treated with SB431542 for 4 weeks, cardiac fibrosis detected by Masson’s trichrome was significantly enhanced, similar to that in afadin cKO hearts 4 weeks after TAC (Fig. 8f,g). These results imply that TGFβ signaling is crucial for the behavior of mononuclear cells in the myocardium and that activation of TGFβ receptor may reduce cardiac damage by limiting myocardial inflammation.

## Discussion

Cardiac IDs incorporate numerous molecular complexes that besides the transmission of electrical and biochemical signals serve as a linkage between cytoskeletons of adjacent cardiomyocytes to provide structural support of always mechanically stressed myocardial tissue. Because of those critical functionalities, disturbance in molecular components of IDs results in cardiomyopathy^10^. In addition, detailed knowledge about the physiology of ID proteins may broaden therapeutical opportunities in clinical cardiology. We explored the significance of an ID protein afadin by analyzing the phenotype of afadin cKO mice. We newly found here that (1) at physiological state, afadin cKO hearts were indistinguishable from the littermate controls; (2) when pressure overload was created by TAC, contrary to controls, afadin cKO mice developed cardiac dysfunction with enhanced fibrosis and apoptosis; (3) early after TAC procedure, macrophage accumulation and expression of MCP-1 were enhanced in afadin cKO hearts; (4) afadin promoted TGFβ receptor-related pathways in the course of chronic pressure overload by TAC, and associated with TGFβ receptor I, which was maximal during the first 2 weeks after TAC; (5) pharmacological antagonism of TGFβ receptor I by SB431542 effectively inhibited Smad2 phosphorylation and amplified macrophage infiltration and collagen synthesis in the myocardium of control mice after TAC. The proposed afadin-mediated signaling in cardiomyocytes for protection against chronic pressure overload is depicted in Fig. 8h.

It is somehow surprising that lack of afadin, a component of the adhesive complexes, does not create obvious cardiac phenotype in the physiological conditions, because it is known that defects of some ID proteins induce cardiomyopathy^10^. The reason for the difference between the previous report and ours might be as follows: (1) the redundancy of available intercellular adhesive complexes developed as protective mechanism to the physiological mechanical load in the heart, and/or (2) differentiation of the functionality of distinctive adhesive complexes, e.g. N-cadherin/catenin, is mainly important for mechanical integrity, but nectin/afadin is more involved in the signaling pathways in cardiomyocytes. Deletion of cardiac N-cadherin, indeed, causes dramatic structural damage of intercalated disks at the basic state^20^. On the other hand, intercalated disks of the hearts lacking nectin are indistinguishable from controls at physiological conditions^12^.

Another novel and interesting discovery in this study is the finding that afadin is an essential mediator of TGFβ receptor-mediated signaling in the heart during chronic pressure overload created by TAC. We detected severe cardiac damage in afadin cKO mice together with inhibition of TGFβ receptor-coupled pathways. TGFβ signaling is known to be diverse and context-dependent^36^, and from our results, the signaling possesses potential cardioprotective effects in relation to afadin action. Indeed, TGFβ may be beneficial in some (patho)physiological contexts in the heart, because it is reported that (1) TGFβ1-null mice die early with diffuse excessive inflammation in many organs including myocardium^37^; (2) Smad2 deletion promotes fibrosis and activation of Smad2 mediates protection against stress-induced cardiac hypertrophy^38^,^39^,^40^; (3) TAK1-deficient hearts display necroptosis, fibrogenesis and progressive cardiac failure^41^. These observations, together with our data, suggest that TGFβ receptor activation might, in addition to mediating detrimental cardiac remodeling, stimulate protective intracellular pathways in cardiomyocytes.

In afadin cKO mice, adverse cardiac remodeling seems to reflect abundant mononuclear infiltration and modulation of TGFβ receptor signaling in the early stages (1–3 weeks) after TAC, which provoked the onset of morphological alterations and LV dysfunction^42^,^43^. These events together with persistent pressure overload could create vulnerable background for deleterious action of various signaling mechanisms (e.g., sympathetic, renin-angiotensin, etc.) that initially compensate chronic heart dysfunction. These mechanisms, however, later result in the induction of adverse effects on cardiac performance and structure^14^,^15^. We provide the finding (Fig. 5g) that triggering mechanism of macrophage accumulation is the result of cardiac afadin deficiency that increased the expression of the macrophage attracting cytokine MCP-1^44^,^45^, immediately after establishment of pressure overload. In the initial stages of cardiac remodeling after TAC, markers of cardiac damage (e.g. apoptosis, Supplemental Fig. S2) are with low intensities, a fact implying that enhanced mononuclear infiltration is not secondary reaction to an early cardiac injury.

Although the immunofluorescence results (Fig. 7c) showed that the expression pattern of afadin (only at IDs) and TGFβ receptor I (both at IDs and on the lateral membrane) was different, the afadin-TGFβ receptor I complex at IDs critically regulated the global TGFβ signaling. Similar mode of regulation at IDs is reported at least for the alpha subunit of cardiac sodium channel (Na_v_1.5) and insulin-like growth factor (IGF)-1 receptor, both of which are expressed in the whole membrane of cardiomyocytes. SAP97, an ID protein, is associated with Na_v_1.5 and silencing of SAP97 in rat cardiomyocytes reduced the Na_v_1.5-mediated sodium current^46^. IGF-1 receptor/PI3-kinase/Akt pathway is downregulated in conditional knockout mice missing vanilloid family type 2 (TRPV2) cation channel, a protein specifically expressed at IDs^47^. In the context of afadin-related protective signaling during pressure overload, our data demonstrate that TGFβ receptor-mediated signaling transduction supported by afadin appears to be the important mechanism in the observed processes of cardiac remodeling, especially in early stages after establishing pressure overload.

As known, cardiac remodeling initiated by pressure overload creates profound alterations in all cardiac cellular and matrix constituents and is triggered by various mechanosensitive and ligand-binding receptors that activate numerous intracellular pathways. Regulation of cardiac remodeling during pressure overload is multi-level machinery that includes interacting signaling networks. Besides TGFβ signaling, there are a number of molecular protective mechanisms acting together with soluble factors, such as ghrelin and adiponectin^48^,^49^. In addition, this study shows that afadin critically functions as a protective molecule against chronic pressure overload-induced cardiac remodeling and heart failure by interacting with TGFβ receptor I to enhance TGFβ-related intracellular signaling.

Impact of afadin deletion in cardiomyocytes on chronic stress-induced remodeling resembles the results in the previous study, which evaluated mice with deletion of nectin-2, a cell adhesion molecule expressed at IDs^12^. Similar to afadin cKO mice, nectin-2 KO mice display aggravated cardiac remodeling under pressure overload. Although both proteins form adhesive complex together^50^, there are clear differences in the pressure overload-associated phenotype and signaling between afadin cKO and nectin-2 KO mice, suggesting distinctive roles of the components of the adhesive complex. In nectin-2 KO hearts, afadin was detected in its usual position at IDs^12^. Nectin-2 deletion resulted in structural defects at IDs, but afadin appears to be not critical for the morphology of IDs during pressure overload (Fig. 2). In addition, the type of knockout of the molecules may also contribute to the difference: the deletion of afadin is limited to cardiomyocytes, while deletion of nectin-2 is global including cardiomyocytes, fibroblasts, endothelial cells, etc.

Mechanosensitivity in the heart at the cardiomyocyte level is multimolecular machinery that involves various proteins located in the cell membrane or in the intracellular compartments^51^. It is crucial mechanism for fine-tuning of cardiac pump performance. Given the fact that afadin, as part of essential for the mechanical integrity structure (IDs), prevents left ventricular dysfunction in afadin cKO mice, it can not be excluded that afadin may influence cardiomyocyte mechano-sensing for the prevention. This might be a limitation of this current investigation.

In summary, afadin is a critical protective molecule at IDs during pressure overload challenge. Our data extend the understanding of sophisticated ID mechanisms preserving cardiac integrity in the course of continuous pressure overload.

## Methods

### Generation of cardiomyocyte-specific afadin cKO mice and establishment of chronic pressure overload by TAC

We crossed C57BL/6 mice homozygous for floxed exon 2 of afadin^7^, and mice expressing Cre recombinase under the control of α-myosin heavy chain gene promoter to create the desired cardiac genotype: homozygous afadin floxed plus Myh6-Cre (afadin cKO). Afadin-floxed littermates without Cre were used as experimental controls. TAC was conducted by tightening 7–0 silk surgical thread around aortic arch with 27-gauge needle to create fixed mechanical obstruction. Heart rate and arterial blood pressure was measured by plethysmographic tail-cuff method (BP-98-AL, Softron), and left ventricular dimensions and pump function were monitored by transthoracic ultrasonography on Vevo 2100 system (VisualSonics Inc). Treatment and experimental procedure of the animals were approved and followed the guidelines of Animal Ethics Committee of the Shiga University of Medical Science, Japan.

### Histological staining of heart sections, immunohistochemistry and transmission electron microscopy

Cryosections were fixed by 4% paraformaldehyde, permeabilized with 0.1% Triton X-100, and blocked with 1% bovine serum albumin. After incubation with primary antibodies (Abs) and fluorescent dye-labeled secondary Abs, confocal images were taken by C1si Laser Scanning Microscope (Nikon). Paraffin sections stained by HE or Masson’s trichrome were analyzed by FXA light microscope (Nikon). Ultrathin sections were prepared as described previously^52^, and observed by transmission electron microscope (H7500, Hitachi).

### Apoptosis assays

To detect apoptotic nuclei in the heart, recombinant terminal deoxynucleotidyl transferase-mediated dUTP nick-end-labeling (TUNEL) reaction was performed using DeadEnd^TM^ Fluorometric TUNEL System (Promega). Activated apoptotic signaling in the cardiomyocytes was assessed by immunostaining for cleaved caspase 3.

### Western blotting and immunoprecipitation

Mouse heart tissue was homogenized mechanically in the lysis buffer. After centrifugation, the supernatants were collected and SDS-PAGE was performed, followed by incubation with primary Abs and horseradish peroxidase (HRP)-labeled secondary Abs. Protein bands were visualized with HRP substrate (Luminata Forte, Millipore Corp.) and analyzed by LAS-4000 (Fujifilm Life Science). For immunoprecipitation, samples were pre-cleared with protein G beads (GE Healthcare Life Sciences) and then incubated with each Ab overnight.

### Statistics

All numerical measurements are presented as mean ± standard error of the mean. Divergences between two groups are examined by Student’s *t*-test. Three or more grouped data are evaluated by one-way analysis of variants followed by Tukey’s *post hoc* test.

## Additional Information

**How to cite this article**: Zankov, D. P. *et al*. Protective effects of intercalated disk protein afadin on chronic pressure overload-induced myocardial damage. *Sci. Rep.* **7**, 39335; doi: 10.1038/srep39335 (2017).

**Publisher's note:** Springer Nature remains neutral with regard to jurisdictional claims in published maps and institutional affiliations.

## Supplementary Material

Supplementary Information
