# Supplementary material for: Protective effects of intercalated disk protein afadin on chronic pressure overload-induced myocardial damage
Source: Sci Rep. 2017 Jan 3;7:39335. doi: 10.1038/srep39335 (PMC5206728; doi:10.1038/srep39335)
Supplement: Supplementary Information [file srep39335-s1.pdf]

Supplementary Information

Protective effects of intercalated disk protein afadin on chronic pressure overload-induced myocardial damage

Dimitar P. Zankov, Akio Shimizu, Miki Tanaka-Okamoto, Jun Miyoshi, Hisakazu Ogita

Supplementary Methods

Generation of cardiomyocyte-specific afadin conditional knockout mice

We crossed C57BL/6 mice homozygous for floxed exon 2 of afadin[1], and mice expressing Cre recombinase under the control of α-myosin heavy chain gene promoter (Myh6-Cre, Jackson Laboratory, USA) to create the desired cardiac genotype: homozygous afadin floxed plus Myh6-Cre (afadin cKO). Mice harboring afadin floxed alleles alone were used as controls. Treatment and experimental procedure of the animals were approved and followed the guidelines of Animal Ethics Committee of the Shiga University of Medical Science, Japan.

Establishment of chronic pressure-overload models by transverse aortic constriction in mice

Mice (age 8 - 11 weeks) were anesthetized with pentobarbital sodium (30 mg/kg intraperitoneally), the trachea was cannulated and ambient air was delivered by rodent ventilator (model 28025, Ugo Basile, Italy) set with tidal volume 0.1-0.2 mL and rate of respiration 120 /min. Transverse aortic constriction (TAC) was introduced between brachiocephalic trunk and left carotid artery by minimally invasive procedure. Aortic arch was exposed through the left second intercostal space and 7-0 silk surgical thread was tightened around 27-gauge needle to create fixed mechanical obstruction with diameter of about 0.4 mm. Sham animals underwent the same treatment except for the ligature. TAC and sham mice were sacrificed after 1 - 8 weeks period of observation according to the experimental protocol.

Hemodynamic measurement and cardiac echography

Heart rate and arterial blood pressure (BP) was measured non-invasively by plethysmographic tail-cuff method (machine model BP-98-AL, Softron, Japan) in conscious animals. Mice were warmed for at least 5 min at 37°C in the cylindrical thermostat of BP-98-AL before and in the course of BP measurement. BP was measured in 2-minute intervals and the mean of 5 steady-state measurements was accepted as actual BP for each animal. Left ventricular (LV)

dimensions and pump function were monitored *in vivo* by transthoracic ultrasonography on Vevo 2100 system (VisualSonics Inc, Canada). During the procedure, mice were anesthetized in supine position on 37°C heating table with isoflurane/air mixture (induction with 4% and maintenance with ~1.5% isoflurane). Two dimensional (B mode) guided M mode measurement of LV parameters was accomplished from parasternal short-axis position at the level of papillary muscles. LV mass (mg) was calculated based on the formula: $1.055 \times [(LVDd + LVPWd + IVSd)^3 - LVDd^3]$, where LVDd substitutes LV diastolic diameter, LVPWd and IVSd: diastolic dimensions of LV posterior wall and interventricular septum, respectively; relative LV wall thickness is calculated as: 2 x LVPWd (mm) / LVDd (mm). Success of TAC operation was confirmed by evaluation of vascular hemodynamics using pulse-wave Doppler mode to measure peak velocity of blood flow in the left and right carotid arteries[2,3].

Histological staining of heart sections and immunohistochemistry

Mice hearts were snap-frozen by liquid nitrogen within a block of water-soluble medium (Surgipath FSC 22, Leica Biosystems, USA), or fixed with 4% paraformaldehyde and subsequently embedded in paraffin blocks. Cryosections, 10 μm thick, were fixed by 4% paraformaldehyde on the top of poly-L-lysine-coated slides immediately after cutting in a cryostat (Leica Biosystems, USA), then permeabilized with 0.1% Triton X-100, and blocked with 1% bovine serum albumin (BSA). Primary antibodies (Abs) were applied in the BSA blocking solution overnight followed by 1 h incubation with fluorescent dye-labeled secondary Abs. Confocal images were taken by C1si Laser Scanning Microscope (Nikon, Japan). Paraffin sections, 4 μm thick, were stained by hematoxylin and eosin (HE) or Masson's trichrome using standard techniques. Images for analysis of myocardial histology were captured by color CCD camera (MicroPublisher 5.0 RTV, Qimaging, Canada) under control of Image-Pro Plus software (Media Cybernetics, USA) mounted on Nikon FXA light microscope. Extent of fibrogenesis in cardiac sections was quantified as ratio of [sum of collagen-positive areas / whole area of visible myocardium x 100 (%)] using Fiji software[4]. Co-localization between afadin and TGFβ receptor I in confocal images is quantified in Fiji software by

calculating values of Pearson correlation and Manders split coefficients of color intensities above the threshold estimated by Costes regression[5,6].

Transmission electron microscopy

Mice myocardium was prepared as described previously[7]. Briefly, mouse myocardium was initially fixed with 4% paraformaldehyde and 2.5% glutaraldehyde, incubated in OsO_4, dehydrated in stepwise increasing concentrations of ethanol, and embedded in epoxy resin. Ultrathin sections mounted on mesh grids were double-stained with 2% uranyl acetate / lead citrate, and observed by Hitachi H7500 transmission electron microscope.

Apoptosis assays

To detect apoptotic nuclei, recombinant terminal deoxynucleotidyl transferase-mediated dUTP nick-end-labeling (TUNEL) reaction was performed on sections of frozen hearts (DeadEnd™ Fluorometric TUNEL System, Promega, USA) and fluorescence of stained nuclei was visualized by confocal microscopy. For each studied group of animals, the percentage of TUNEL-positive nuclei is estimated after evaluation of 1000 nuclei derived from 3 animals. Activated apoptotic signaling in the cardiomyocytes was assessed by immunostaining of frozen cardiac sections for cleaved caspase 3 and area of positive staining is compared among all groups (3 animals per group).

Western blotting and immunoprecipitation

Mouse heart tissue was homogenized mechanically in the lysis buffer containing 320 mmol/L sucrose, 20 mmol/L Tris-HCl (pH 7.5), 2 mmol/L EDTA, 0.1% Triton X-100, and supplied with protease inhibitors (20 μg/mL aprotinin, 20 μg/mL leupeptin, 1 mmol/L PMSF). After centrifugation, the supernatants were collected as protein samples. SDS-PAGE was performed with 30 μg protein per lane followed by blotting on PVDF membrane using Mini PROTEAN II and Mini Trans-Blot cells (Bio-Rad Laboratories, USA). Primary Abs were incubated overnight in 5% skim milk or 5% BSA solution at 4°C. Horseradish peroxidase (HRP)-labeled

secondary Abs were applied for 1 h in 5% skim milk. To visualize the protein bands, PVDF membrane was treated with HRP substrate (Luminata Forte, Millipore Corp., USA) for 1 - 5 min and observed on luminescent image analyzer LAS-4000 (Fujifilm Life Science, Japan). The ratio between phosphorylated and total protein molecule of interest was estimated on the same PVDF membrane: first, primary Ab against phosphorylated protein was probed, then membrane was stripped (WB Stripping Solution, Wako Chemicals, Japan), blocked again with skim milk, and Ab against the total protein was applied. Protein samples for immunoprecipitation, 300 µg each, were pre-cleared with protein G beads (GE Healthcare Life Sciences, UK), incubated with 1.5 µg anti-transforming growth factor (TGF) β receptor I, 2 µg anti-afadin or 2 µg anti-GFP Ab for immunoprecipitation overnight, and finally protein G beads were separated from Ab/protein complex in SDS-PAGE sample buffer heated at 95°C. For detection, Western blots using primary Abs against the both proteins of interest were performed.

Antibodies for Western blotting, immunoprecipitation and immunostaining

<u>*Primary Abs and dilutions*</u>: anti-l-afadin rabbit polyclonal – 1:1000 (Sigma, USA), anti-N-cadherin mouse monoclonal – 1:200 (BD Biosciences, USA), anti-desmoglein 2 rabbit monoclonal – 1:200, anti-F4/80 rabbit monoclonal – 1:200 (Abcam, UK), anti-Smad2 and phospho-Smad2 (Ser465/467) rabbit monoclonal – 1:1000, anti-Akt and phospho-Akt (Ser473) rabbit monoclonal – 1:1000, anti-TAK1 and anti-phospho-TAK1 (Thr184/187) rabbit monoclonal – 1:1000, anti-caspase 3 and anti-cleaved caspase 3 rabbit monoclonal – 1:400, anti-GAPDH hybridoma monoclonal – 1:1000 (MBL), anti-transforming growth factor (TGF)β receptor I rabbit polyclonal – 1:200 (Santa Cruz Biotechnology, USA), anti-GFP mouse monoclonal, clone mFX75 – 1:500 for Western blotting, clone mFX73 – 2 µg for immunoprecipitation (Wako Chemicals, Japan).

<u>*Secondary Abs, fluorescent dyes, and dilutions*</u>: Alexa Fluor 488 or 555 goat anti-rabbit – 1:1000, Alexa Fluor 488 or 555 goat anti-mouse – 1:1000 (Invitrogen, USA), wheat germ agglutinin (WGA) tetramethylrhodamin conjugate – 5 µg/mL, rhodamin-phalloidin – 5 µg/mL

(Invitrogen), DAPI – 1 μg/mL (Dojindo, Japan), propidium iodide – 1 μg/mL (Invitrogen), HRP-linked goat anti-rabbit IgG or anti-mouse IgG Abs – 1:2000 (Cell Signaling Technology, USA).

Transfection into COS7 cells

Total RNA, extracted from mouse Sarcoma180 cells, was reverse-transcribed, and mouse afadin cDNA was subcloned using the multicloning site of pCR3.1 vector (Invitrogen). Then, the cDNA was cut out and inserted into pEGFP-C3 vector (Clontech, USA) to make a plasmid (pEGFP-afadin) that induces the expression of GFP-tagged mouse afadin protein in mammalian cells. COS7 cells (70-90% confluent) were transfected with pEGFP-afadin plasmid using Lipofectamin 2000 (Invitrogen) according to the manufacturer's instructions. At 48h after the transfection, protein samples are prepared in RIPA buffer (135 mmol/L NaCl, 50 mmol/L Tris-HCl (pH 7.4), 0.1% sodium dodecyl sulfate, 0.5% sodium deoxycholate, 1% Nonidet P-40) using standard protocol.

Block of TGFβ receptor I in TAC-operated control mice *in vivo*

Dimethyl sulfoxide (DMSO, Wako Chemicals, Japan) or SB431542 (Cayman Chemical, USA), a selective inhibitor of TGFβ receptor I (ALK5), was injected intraperitoneally in TAC-operated control mice daily for the period of 2 or 4 weeks. SB431542 was applied 0.2 mg/kg dissolved in 100 μL DMSO; 100 μL DMSO was injected in some TAC mice as negative control.

Quantification of MCP-1 mRNA in cardiomyocytes

TAC operation was performed in control and afadin cKO mice. Three days later, the mice were sacrificed and small chunks of myocardium were digested by 0.5 mg/ml collagenase type 2 (Calbiochem, USA) at 37°C for 20-25 min in the solution containing: 130 mmol/L NaCl, 5.4 mmol/L KCl, 0.5 mmol/L $MgCl_2$, 0.33 mmol/L NaH_2PO_4, 22 mmol/L glucose, 25 mmol/L HEPES, 0.2 mmol/L $CaCl_2$, and 0.1% BSA. After trituration, cardiomyocytes were separated

from the debris and total RNA was extracted using RNeasy kit (Qiagen, Germany). cDNA was obtained by reverse transcription PCR (ReverTra Ace^{TM} qPCR RT Master Mix, Toyobo, Japan) with random primer. For quantitative PCR the samples were prepared using Light Cycler 480 SYBR Green I Master (Roche, Germany) and amplified in Roche Light Cycler 480 with primers: MCP-1 forward 5'-GCCCCACTCACCTGCTGCTACT-3', MCP-1 reverse 5'-CCTGCTGCTGGTGATCCTCTTGT-3'. Relative quantity of MCP-1 mRNA was normalized to β-actin in the same samples. MCP-1 / β-actin ratio of C_P was used for comparison.

Statistics

All numerical measurements are presented as mean ± standard error of the mean. Divergences between two groups are examined by Student's *t*-test. Three or more grouped data are evaluated by one-way analysis of variants followed by Tukey's *post hoc* test. Data are presented as mean ± S.E.M.

Supplementary References

1. Majima, T. *et al.* Involvement of afadin in the formation and remodeling of synapses in the hippocampus. *Biochem. Biophys. Res. Commun.* **385**, 539-544 (2009).
2. Collins, K. A., Korcarz, C. E. & Lang, R. M. Use of echocardiography for the phenotypic assessment of genetically altered mice. *Physiol. Genomics.* **13**, 227-239 (2003).
3. Liao, Y. *et al.* Echocardiographic assessment of LV hypertrophy and function in aortic-banded mice: necropsy validation. *Am. J. Physiol. Heart Circ. Physiol.* **282**, H1703-8 (2002).
4. Schindelin, J. *et al.* Fiji: an open-source platform for biological-image analysis. *Nat. Methods.* **9**, 676-682 (2012).
5. Costes, S. V. *et al.* Automatic and quantitative measurement of protein-protein colocalization in live cells. *Biophys. J.* **86**, 3993-4003 (2004).
6. Dunn, K. W., Kamocka, M. M. & McDonald, J. H. A practical guide to evaluating colocalization in biological microscopy. *Am. J. Physiol. Cell Physiol.* **300**, C723-742 (2011).
7. Komura, H. *et al.* Establishment of cell polarity by afadin during the formation of embryoid bodies. *Genes Cells.* **13**, 79-90 (2008).

Supplementary Figure Legends

Supplementary Figure S1. Cardiac phenotype of afadin cKO mice. (**a**) Western blots demonstrating the degree of afadin expression in 5 organs taken from control and afadin cKO mice. (**b**) Co-immunostaining of afadin (green) and actin filaments (red) in cardiac sections of control and afadin cKO mice. White arrows: positive for afadin at IDs, Yellow arrowheads: positive for afadin in non-cardiomyocytes. (**c**) HE-stained cardiac sections of control and afadin cKO mice.

Supplementary Figure S2. Time course of apoptotic markers after TAC operation in control and afadin cKO hearts. (**a**) Confocal images showing TUNEL-positive apoptotic nuclei (green) among the scanned nuclei stained with propidium iodide (red) in the heart sections before (0 week), 1, 2 and 3 weeks after the TAC procedure. Yellow arrows: TUNEL-positive cells. (**b**) Immunostaining of cleaved caspase 3 (green) before, 1, 2 and 3 weeks after TAC. Wheat germ agglutinin (WGA: red) staining for the surface membrane of cardiomyocytes, and DAPI (blue) staining for the nuclei. Images below the respective photos showing magnification of depicted rectangular areas. (**c** and **d**) Summary graphs for percentage of TUNEL-positive nuclei (**c**) and cleaved caspase 3-positive area (**d**) before, 1, 2 and 3 weeks after TAC. Data presenting at 8 weeks after TAC are derived from Fig. 3.

Supplementary Fig. 1

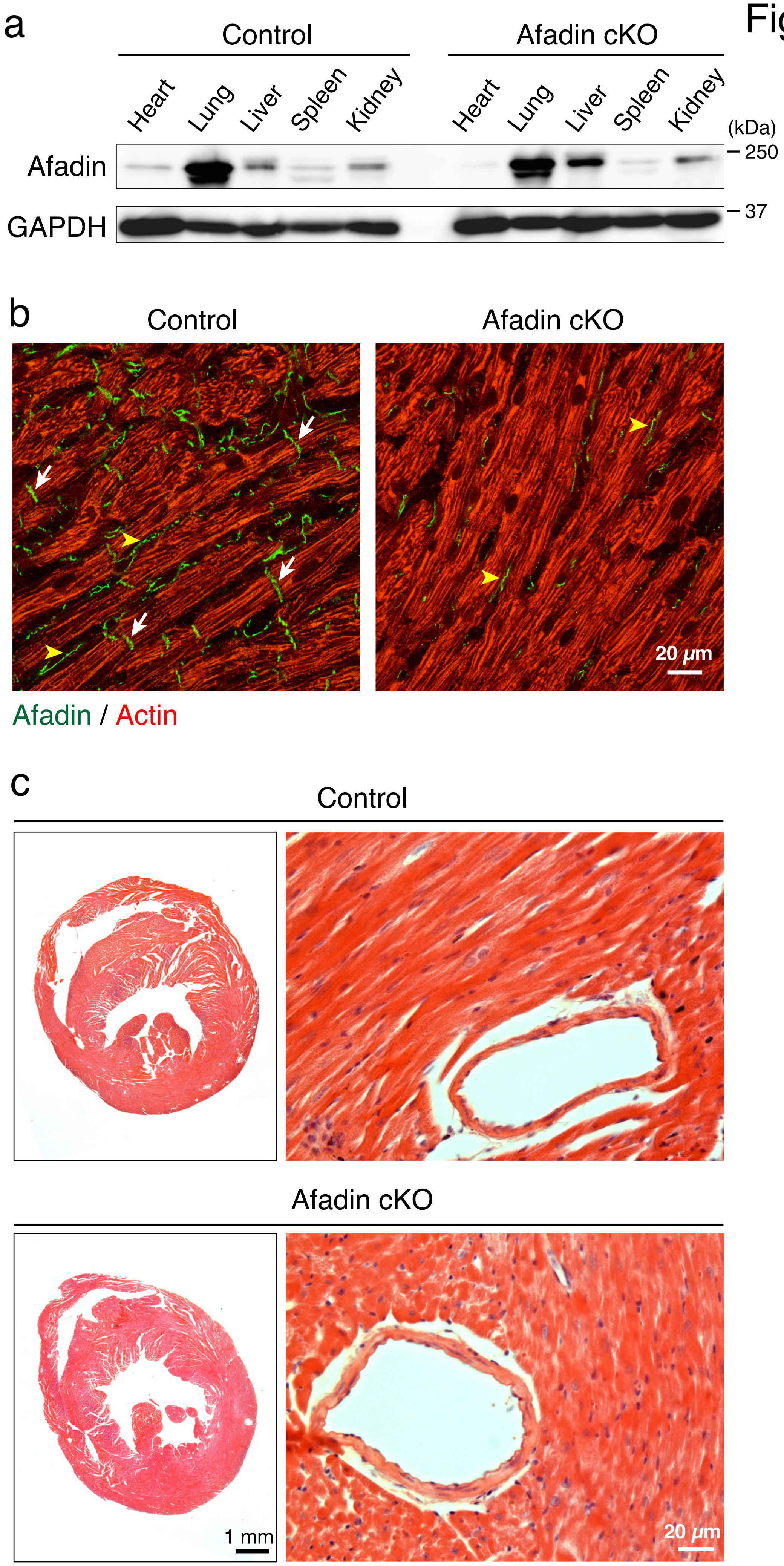

Supplementary Fig. 2

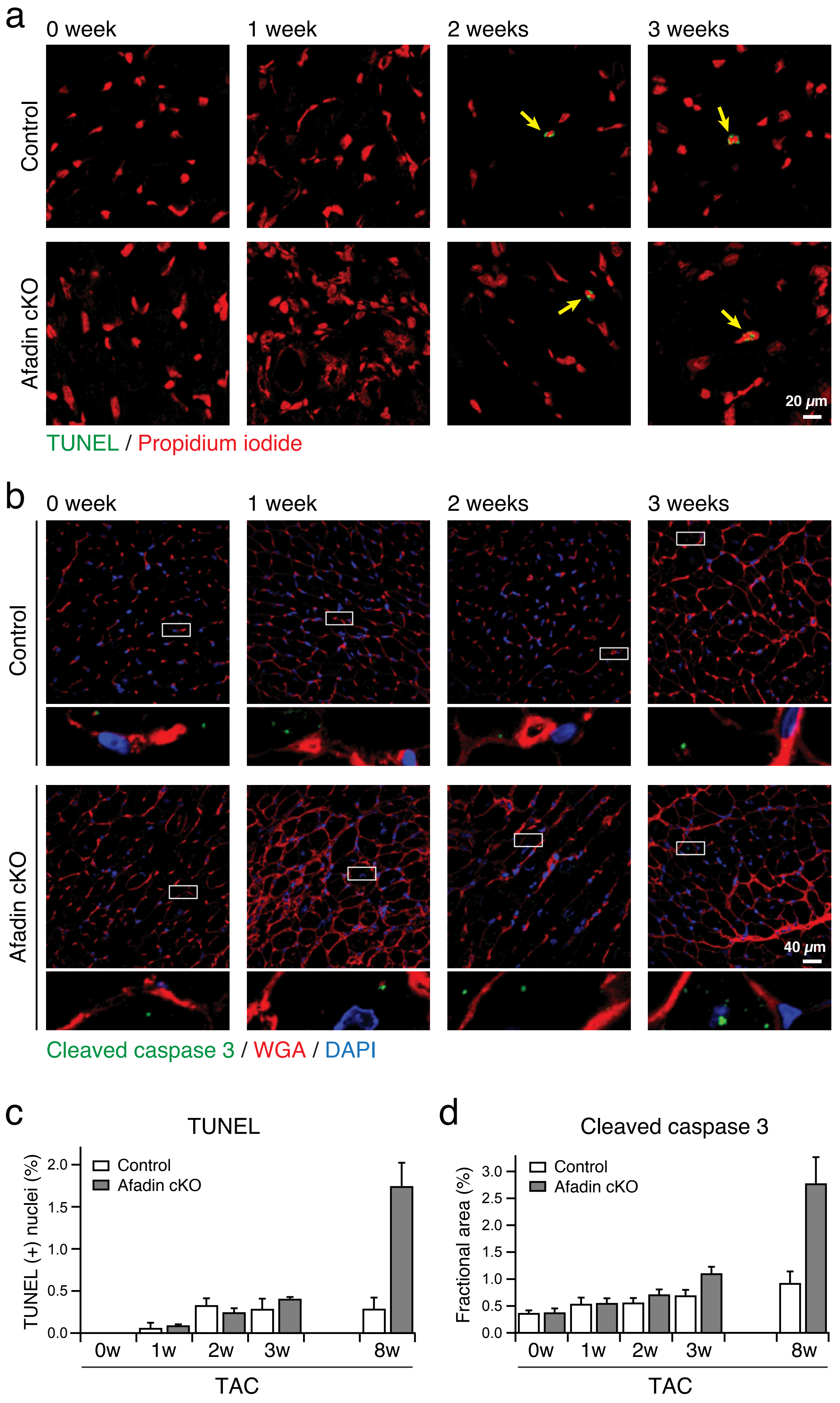

Supplementary Table 1

Hemodynamic measurements

	Control (n = 8)	Afadin cKO (n = 8)
Heart rate (bpm)	671 ± 5.9	678 ± 8.5
Systolic BP (mmHg)	112.7 ± 1.2	106.0 ± 2.0
Diastolic BP (mmHg)	70.3 ± 2.0	71.1 ± 1.9

Echocardiographic measurements

	Control (n = 8)	Afadin cKO (n = 8)
LVPWd (mm)	0.74 ± 0.02	0.69 ± 0.02
IVSd (mm)	0.70 ± 0.02	0.67 ± 0.02
LVDd (mm)	3.65 ± 0.19	3.68 ± 0.11
LV mass (mg)	88.5 ± 5.5	83.3 ± 4.6
EF (%)	65.8 ± 3.6	65.5 ± 4.2

BP: blood pressure, LVPWd, IVSd and LVDd: left ventricular diastolic dimension of posterior wall, ventricular septum and cavity, respectively, EF: ejection fraction.
